# Capturing Fast Gas Migration in Proteins

**DOI:** 10.3390/molecules31183148

**Published:** 2026-09-08

**Authors:** Suk Min Kim, Mohd Faheem Khan

**Affiliations:** 1 Department of Biotechnology, The Catholic University of Korea, 43 Jibong-ro, Wonmi-gu, Bucheon-si 14662, Gyeonggi-do, Republic of Korea; 2 UCD School of Agriculture and Food Science, University College Dublin, Belfield, D04 V1W8 Dublin, Ireland

**Keywords:** gas diffusion, molecular dynamics, ligand migration, time-resolved crystallography, protein cavities, molecular transport

## Abstract

Small gases pose an unusual problem for studies of molecular transport in proteins. O_2_, CO, H_2_, and NO can cross short-lived internal spaces opened by protein fluctuations, often faster than experiments can follow continuous migration. Time-resolved crystallography can localize sufficiently populated intermediates, whereas spectroscopy, isotope exchange, and kinetic measurements report molecular exchange over their respective timescales without resolving the complete route. Pressurized noble-gas structures expose internal accommodation sites but rely on surrogate molecules whose size and interactions differ from those of physiological gases. Geometry-based tunnel searches identify available space, while molecular dynamics follows explicit movement through a fluctuating protein. Free-energy and enhanced-sampling approaches can access states or transitions that remain undersampled in direct trajectories. These techniques resolve different quantities rather than progressively more accurate estimates of gas transport. In this Perspective, we argue that gas-migration pathways should be evaluated by the physical consistency of independent observables, with each method interpreted according to the quantity it resolves. This distinction explains why a cavity visible crystallographically may not carry substantial flux, why a rapidly crossed route can remain structurally inconspicuous, and why static narrowing can alter diffusion without predicting its magnitude. Agreement among methods can support a transport assignment when the quantities they resolve are physically consistent with the same mechanism; apparent disagreement may instead reflect differences among occupancy, accessibility, residence, energetic preference, and molecular traffic.

## 1. Introduction

Gas molecules present an unusual case in protein transport because the molecular event of interest can be easier to perform than to observe. O_2_, CO, H_2_, and NO require little free volume and can pass through internal cavities connected by modest side-chain or backbone motion. Fast microscopic passage is not the same as fast overall transport. An individual crossing event can be very rapid while a rare gating fluctuation elsewhere in the protein still limits the net rate. In this context, fast refers specifically to this microscopic step rather than the net process. Its absolute timescale depends on the protein, gas species, and molecular event. Work on enzyme gates, buried active sites, tunnel engineering, and gas-processing metalloenzymes has established that these internal routes can influence substrate access and reaction behavior [1,2,3,4,5]. Internal openings may be persistent, intermittent, or distributed across several cavities, with the relevant geometry depending on the molecular species and protein state [6]. The resulting challenge is to reconstruct rapid molecular movement from measurements with different spatial and temporal resolution and molecular specificity. Early oxygen-quenching experiments already showed that dioxygen can penetrate protein interiors at rates difficult to reconcile with a permanently sealed structural picture [7].

**Figure 1:**
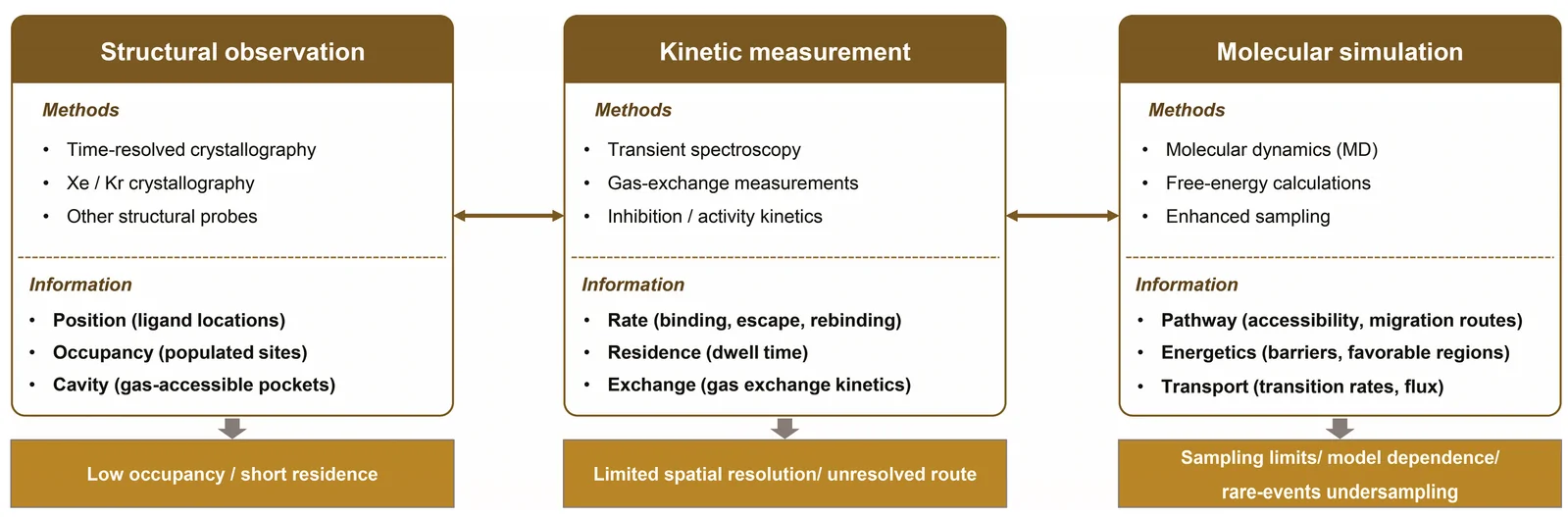
Different observational views of fast gas migration in proteins. Structural observations identify ligand positions, occupied sites, and gas-accessible cavities. Kinetic measurements quantify binding, escape, residence, and exchange, whereas molecular simulations resolve pathway accessibility, energetic barriers, and, depending on the calculation, transport rates. The lower boxes indicate the principal limitation of each approach; none of these observables can substitute for another. The temporal range depends on the specific method and implementation and is therefore not represented by a common timescale.

Structural studies locate internal cavities, kinetic measurements resolve binding and exchange, and simulations reconstruct otherwise unresolved molecular motion (Figure 1, Table 1). Apparent disagreement among these methods can arise because they resolve different aspects of transport, not because one of them is wrong. The difficulty is deciding how these distinct observables should be weighed when assigning a gas-transport pathway. Our central argument is that gas-migration pathways should not be assigned from individual structural, kinetic, or computational observations alone, but from independent observables that are physically consistent with the same transport mechanism.

## 2. Experimental Views of Fast Gas Motion

### 2.1. Structural Observation Depends on Molecular Residence

Time-resolved crystallography provides perhaps the closest experimental equivalent to a molecular movie when a suitable reaction trigger and sufficient ligand population are available. Cryogenic and time-resolved structural work on myoglobin established that photodissociated CO can occupy internal cavities while the protein undergoes coupled conformational relaxation [8,25,26,27]. In the room-temperature Laue experiment of Šrajer and colleagues, CO photolysis was triggered by a 7.5 ns laser pulse, structural and ligand changes were followed from approximately 1 ns to 1.9 ms, and CO docking-site populations of approximately 10% were detectable under these experimental conditions [26]. Mutational and kinetic mapping showed that the distal His64 region is a major route for ligand entry and escape, while internal xenon cavities act as secondary migration sites [12]. More recently, time-resolved serial femtosecond crystallography followed CO dynamics after flash photolysis in cytochrome c oxidase, resolving transient ligand and active-site structural changes [28]. Temporal resolution alone does not determine whether a transient state can be resolved. Trigger efficiency, synchronization, signal quality, temperature, and structural heterogeneity can also affect the observed electron density. A site becomes structurally visible when enough molecules remain there long enough to produce measurable density. A region crossed rapidly may contribute to transport while remaining weakly populated or invisible.

### 2.2. Kinetics Detects Movement That a Structure May Not Locate

Transient spectroscopy, flash photolysis, isotope exchange, and electrochemical kinetics probe gas binding, release, and exchange. They can resolve binding, dissociation, rebinding, and exchange over broad time ranges without reconstructing the complete atomic path. Xenon competition experiments in myoglobin showed that occupation of internal cavities can alter rebinding and escape kinetics, demonstrating that kinetic perturbation can report on otherwise hidden migration states [13]. Models that couple protein relaxation to ligand migration likewise reproduce complex rebinding behavior over wide time and temperature ranges without requiring direct structural observation of every intermediate [14]. Hydrogenase studies illustrate this distinction. Protein film voltammetry and isotope-exchange measurements established quantitative rates for CO and H_2_ exchange [15], and subsequent tunnel mutations reduced gas diffusion by orders of magnitude without a simple proportional relation between static obstruction and measured transport [16]. This mismatch is consistent with transient passage through conformations that appear closed in a crystallographic snapshot. Independent support comes from magnetic relaxation dispersion NMR measurements of buried water exchange. Benchmarked against a millisecond-scale simulation, they show that rare structural fluctuations, not persistent openings, govern access to a protein interior [29].

### 2.3. Pressurized Probes Map Accommodation Rather than Complete Traffic

Xe and Kr partition into hydrophobic protein cavities and yield strong diffraction contrast, making otherwise empty internal spaces visible. The classic xenon structure of metmyoglobin resolved four internal sites at 1.9 Å [9]. In myoglobin, those sites overlap with regions favored by O_2_, CO, and NO in computational free-energy maps [21]. In xenon-pressurized carbon monoxide dehydrogenase/acetyl-CoA synthase, 17 of 19 Xe sites fell within regions predicted by a cavity calculation, showing agreement between an experimental probe and a computed internal channel [10]. The same probe strategy extends to hydrogenases as well. Krypton derivatization of an O_2_-tolerant membrane-bound [NiFe] hydrogenase revealed an extended hydrophobic gas network connecting the surface to the buried active site, showing that pressurized noble-gas probes can map extensive internal channel systems across different protein folds [11]. The probe remains a surrogate. Xe is larger and more polarizable than physiological diatomic gases, while H_2_ is smaller. Xenon occupancy therefore establishes that a cavity can accommodate a gas-like probe. It does not establish that every physiological molecule uses that cavity with the same frequency, and absence of xenon cannot exclude a narrow or short-lived passage accessible to a smaller molecule.

## 3. Computational Analysis of Gas Migration

### 3.1. Static Pathway Analysis Identifies Available Space

Geometry-based algorithms reduce protein interiors to reproducible descriptions of cavities, pores, bottlenecks, and connected pathways. CAVER 3.0 extended tunnel detection to dynamic protein ensembles [17], and MOLE 2.0 provided a complementary description of biomacromolecular channels [18]. Related tools extended this approach to large-scale channel annotation, web-based analysis, axis-based geometry, and docking-linked energetics [30,31,32,33]. These methods identify candidate routes and permit comparisons across structures. An algorithmically identified tunnel describes geometric connectivity and steric accessibility in the structures supplied to the analysis, but does not establish gas occupancy, molecular flux, or functional pathway use.

### 3.2. Molecular Dynamics Reveals Routes That Structures Cannot Retain

Explicit molecular dynamics allows the gas and surrounding protein to evolve together. Simulations of CpI [FeFe] hydrogenase showed that H_2_ and O_2_ can exploit packing defects that open and close during protein fluctuations, revealing access behavior that cannot be inferred from one crystal structure [19]. Similar work on bacterial H-NOX proteins found that CO, NO, and O_2_ do not use identical internal routes or residence patterns even among closely related protein folds [34]. Studies of oxygen-dependent enzymes reach the same conclusion. In 12/15-lipoxygenase, computation identified dynamic oxygen-access channels that were tested by mutagenesis and oxygen-dependent kinetics [35]. A more recent study of the same enzyme family shows that this channel network is not a fixed protein property. Substrate binding and membrane association reshape which of two identified O_2_ tunnels is used, altering the balance of access between them [36]. In flavoenzymes, enhanced-statistics simulations combined with mutagenesis, rapid kinetics, and crystallography supported several funnel-shaped O_2_ routes converging on the reactive flavin site [20]. The visual completeness of a molecular trajectory should not be confused with statistical completeness. Gas movement can be sampled within one basin while slower gating or hydration changes remain underexplored. An observed passage therefore establishes a possible route, not its physiological contribution to flux. Route recurrence across independent trajectories provides stronger evidence than a single crossing, although quantitative flux requires adequate sampling of the relevant conformational states and transitions. Gas migration predicted by MD also depends on the force-field description of gas–protein interactions. Fixed-charge models do not explicitly account for electronic polarization, while polarizable force fields or QM/MM approaches can be considered when polarization or active-site electronic interactions substantially affect gas energetics [37,38].

### 3.3. Sampling Methods Extend Observation but Change the Observable

Long molecular-dynamics trajectories create their own post-processing problem, since tunnel ensembles can contain hundreds of thousands of snapshots; TransportTools and a related divide-and-conquer strategy cut this cost without changing the resulting tunnel clusters [39,40]. A related efficiency gain comes from using multiple non-interacting gas copies within one evolving protein trajectory [41]. This improves sampling of gas positions and possible migration routes at a fraction of the computational cost, without equivalent independent sampling of protein conformations. These trajectory-analysis and accelerated positional-sampling strategies do not by themselves yield transport rates. Free-energy calculations instead describe an energetic landscape, mapping favorable regions for O_2_, CO, NO, and Xe without requiring explicit diffusion events [21], or reconstructing a network of minimum-free-energy routes that shows a dominant gate is important but not exclusive [22]. The choice of approach depends on the quantity of interest. Enhanced sampling can improve exploration of rare states or migration events, while replica-based simulations can broaden conformational sampling and transition-path methods can characterize transitions between defined states [23]. Convergence should be assessed using independent sampling and the stability of state populations, free-energy profiles, or transition statistics. For biased simulations, the dependence of these quantities on the applied bias should also be examined. Free-energy maps alone do not provide transport rates. When bias is introduced to accelerate sampling, raw event frequencies cannot be interpreted directly as kinetic rates. State-based kinetic models can estimate rates from adequately sampled transitions between defined states [24,42]. In myoglobin, this approach assigned approximately 90% of the calculated solvent-to-distal-pocket CO flux to the His64 gate [24], consistent with earlier simulations in the crystallographic environment [43].

## 4. Interpreting Gas Migration Across Observables

### 4.1. Visibility, Occupancy, and Molecular Traffic Are Not Equivalent

Structural visibility depends on population. High occupancy can arise because molecules enter a cavity frequently, because they leave it slowly, or through both effects. The same ambiguity is present in simulated density maps. A high-occupancy internal pocket can be a productive intermediate or a trap, while a rapidly crossed passage can have low occupancy despite contributing to molecular traffic. Crystallographic docking sites, xenon cavities, mutationally mapped entry routes, and simulated free-energy minima can overlap only partially [8,12,13,21,22,25,26,27]. Low structural occupancy therefore does not exclude a kinetically important route. We consider structural accessibility sufficient to identify a candidate route, but not to establish its contribution to transport. Pathway assignment requires independent observables that are physically consistent with the same transport mechanism.

### 4.2. Agreement Can Support Pathway Assignments, While Exceptions Remain Informative

**Figure 2:**
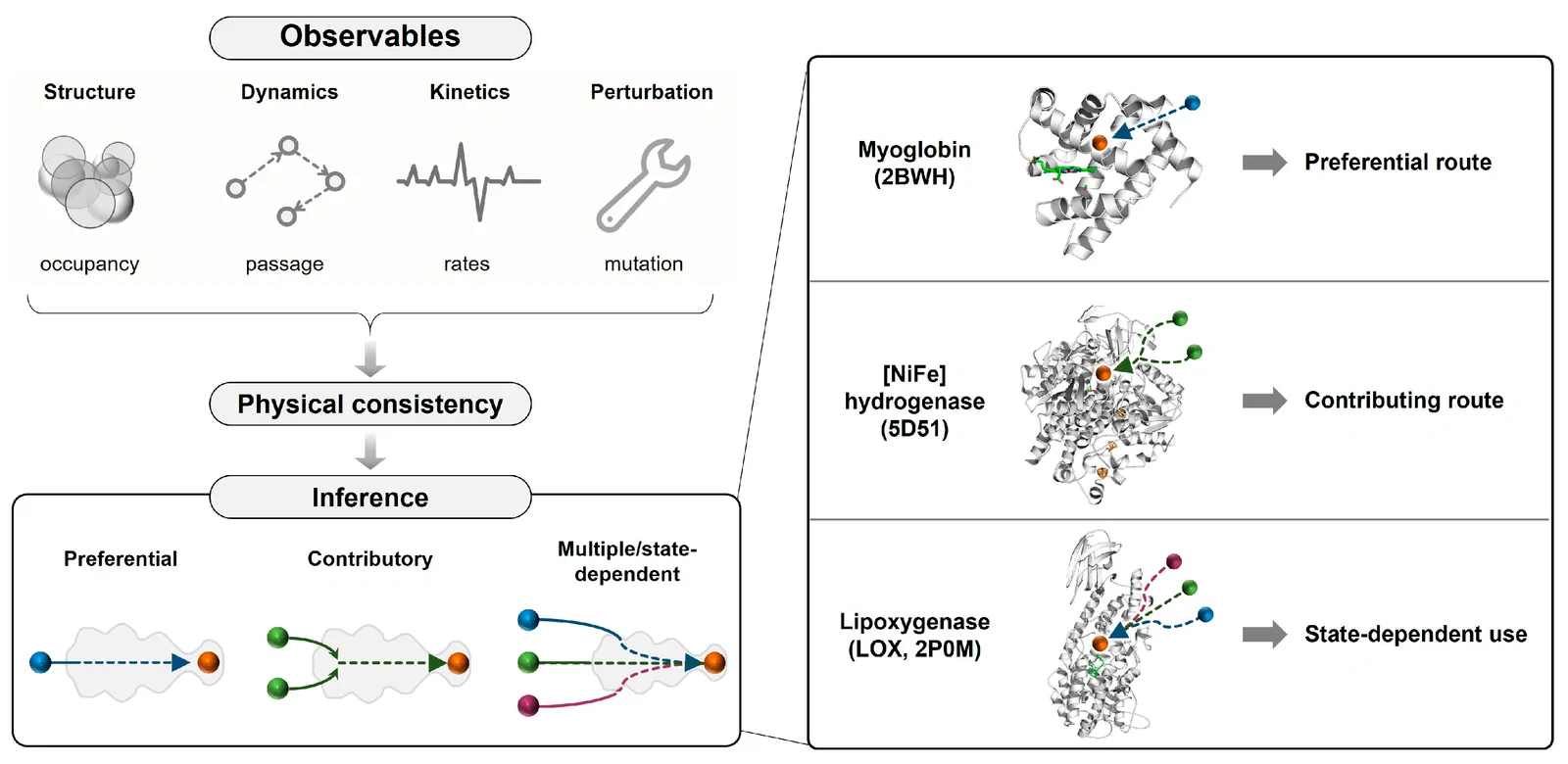
From complementary observations to gas-pathway inference. Structural occupancy, dynamic passage, kinetic rates, and perturbation provide complementary evidence whose physical consistency supports pathway inference. Representative proteins are myoglobin, [NiFe] hydrogenase, and lipoxygenase (PDB 2BWH, 5D51, and 2P0M, respectively). Pathway arrows are schematic (based on the experimental and computational evidence, not directly resolved trajectories). In the inference box, arrows indicate direction, dashed lines indicate alternative routes, and colors distinguish different pathways.

Representative protein structures in Figure 2 illustrate how structural, dynamic, kinetic, and perturbational evidence can be considered together when evaluating gas-migration pathways. Myoglobin provides a well-documented case of convergence. Time-resolved crystallography, ligand rebinding experiments, explicit simulations, free-energy calculations, and kinetic models now support a dominant role for the distal histidine gate while retaining secondary internal routes [12,13,22,24,26,43]. This convergence is not universal. Flavoenzymes can use several O_2_ access pathways [20], and lipoxygenase shows how a dynamically connected route can become functionally important without behaving as a permanently open tube, and can shift with substrate and membrane context [35,36]. Gas-sensing H-NOX proteins further show that similar overall folds do not guarantee identical migration behavior for different gases [34]. Multiscale simulation of the hypoxia-sensing enzyme PHD2 identified a well-defined hydrophobic tunnel for O_2_ delivery and produced rate constants consistent with the enzyme’s slow reaction with dioxygen [44]. Subsequent tunnel engineering of PHD2 altered gas-association kinetics and catalytic efficiency, providing experimental support for the functional contribution of this route [45]. Gas transport can involve a dominant gate, several competing entrances, or transiently connected cavity networks, with pathway use changing with gas identity and protein state. Such differences may reflect variation in transport behavior rather than methodological inconsistency.

### 4.3. When Disagreement Is Physically Meaningful

Static narrowing can identify the correct region of resistance while failing to predict the magnitude of a rate change because ordinary protein fluctuations partly restore passage that a crystal structure shows as closed [15,16]. Such discrepancies should not automatically be assigned to differences in the measured quantity. Incompatible conditions, inadequate sampling, force-field error, and incomplete structural models remain alternative explanations that must be excluded. Only once these possibilities are ruled out does a discrepancy become informative about the transport process itself, rather than about the limits of one measurement. The lipoxygenase and flavoenzyme studies linked computed access behavior to mutation-dependent kinetics [20,35], while simulation, mutagenesis, and xenon-binding data converged on the same O_2_ access regions in a cofactor-independent dioxygenase [46]. Agreement is informative when independent measurements converge on the same transport behavior under compatible conditions and timescales.

## 5. Conclusions

Fast diffusion is not, by itself, what makes gas migration hard to observe. The difficulty arises because passage can be brief and occupancy too low for transport to appear as a persistent structural event. A route assignment therefore requires evidence that is physically consistent across independent measurements rather than a simple count of methods in agreement. Gas transport varies with protein dynamics, internal geometry, and the properties of the migrating molecule. A route can be structurally inconspicuous yet kinetically important, just as a high-occupancy cavity may contribute little to net molecular traffic. Claims about a gas pathway should specify which observable actually supports it, rather than treat the pathway as a single structural entity waiting to be discovered. We see three priorities for future studies. First, occupancy, dynamics, and transport rates should be compared under closely matched molecular conditions. Second, predicted gates or bottlenecks should be perturbed experimentally to test their causal contribution to transport. Third, the quantitative relationship between occupancy, residence, and flux needs to be established across experimental and computational observations.

## Figures and Tables

**Table 1:** Approaches for resolving gas migration in proteins.

Category	Method	Observable	Inference	Limitation	Complementary Approach	Ref.
Structural observation	Time-resolved crystallography	Electron density, time-dependent occupancy, structural change	Populated ligand sites, structural relaxation	Low-occupancy or short-lived states missed	Spectroscopy, MD	[8]
	Xe/Kr crystallography	Noble-gas occupancy	Gas-accessible cavities, internal voids	Surrogate occupancy may not reflect native-gas transport	Native-gas kinetics, MD	[9,10,11]
Kinetic measurement	Transient spectroscopy	Rebinding, escape, exchange rates	Migration timescale, kinetic partitioning	Atomic route unresolved	Structural mapping, mutagenesis, simulation	[12,13,14,15]
	Activity/ Inhibition kinetics	Reaction or inhibition rate	Functional gas accessibility	Transport and catalysis may be kinetically coupled	Targeted mutations, independent transport measurements	[16]
Molecular simulation	Geometry analysis	Connectivity, radius, bottleneck, length	Candidate tunnels, steric constraints	Dynamics, energetics, flux absent	Structural ensembles, MD	[17,18]
	Explicit MD	Atomic trajectories, passage events	Dynamic routes, transient gates	Rare events, slow-state undersampling	Replicates, longer trajectories, enhanced sampling	[19,20]
	Free-energy calculations	Free-energy minima, barriers	Preferred sites, barrier hierarchy	Transport rate not directly determined	Transition sampling kinetic modeling	[21,22]
	Enhanced/ Transition sampling	Rare-transition sampling, pathway ensembles	Rare routes, broader configurational sampling	Bias and Coordinate dependence	Convergence tests, unbiased validation	[23]
	Kinetic modeling	Transition probabilities, rate constants	State populations, competing routes, transport rates	State-definition and sampling dependence	Experimental constraints, independent trajectories	[24]

Structural methods resolve populated or gas-accessible sites, kinetic measurements provide temporal or functional information, and molecular simulations describe pathways, energetics, or transport kinetics according to the calculation used. Operational terms used in this Perspective are defined as follows: occupancy, the fraction or population of molecules at a site; accessibility, the ability of a molecule to reach a site or connected region; residence, time spent at a site; energetics, the free-energy preference or barrier associated with a site or transition; flux, molecular traffic through a route per unit time; and transport rate, the rate of transfer between defined states or regions. Temporal range and detectability are method- and implementation-dependent. Quantitative values are given where supported by specific studies. MD, molecular dynamics; Xe, xenon; Kr, krypton.

## Data Availability

No new data were created or analyzed in this study. Data sharing is not applicable to this article.

## Abbreviations

The following abbreviations are used in this manuscript:

MD	Molecular dynamics
PHD	Prolyl hydroxylase
